# The association between increases in nitrate in drinking water and colorectal cancer incidence rates in California, USA

**DOI:** 10.1007/s10552-025-02003-5

**Published:** 2025-04-28

**Authors:** Ricardo Cisneros, Marzieh Amiri, Hamed Gharibi

**Affiliations:** 1 Health Sciences Research Institute, University of California Merced, 5200 North Lake Road, Merced, CA 95343 USA; 2 Shahroud University of Medical Sciences, Shahroud, Iran

**Keywords:** Colorectal cancer, Nitrate, Disinfection-by-products, Drinking water, California

## Abstract

**Purpose:**

The water resources in California are polluted with nitrate (NO_3_) due to the ever-increasing application of nitrogen-based fertilizers. Considering the potential connection between NO_3_ in drinking water and the incidence rate of colorectal cancer, this study aims to investigate the association between long-term exposure to NO_3_ via drinking water and the incidence of colorectal cancer from 2010 to 2015 in California.

**Methods:**

A total of 56,631 diagnoses of colorectal cancer were recorded from 2010 to 2015. A generalized linear model was used to obtain the risk ratio (RR) and 95% confidence interval associated with a 1 mg/l-NO_3_ increase in NO_3_ concentration across five latency periods. The potential effect modification by sex, race/ethnicity, and age (> 40, 41–64, 65–90, and > 90) was explored through stratification.

**Results:**

The association between increases in the concentration of NO_3_ at lag 0–1, lag 0–5, lag 0–10, lag 0–15, and lag 0–20 (RRs: 1.056 [1.055, 1.058]; 1.066 [1.063, 1.069]; 1.030 [1.028, 1.031]; 1.017 [1.016, 1.018]; 1.035 [1.034, 1.037], respectively) was positively associated with the RR of colorectal cancer. Sex was not found to be a significant modifier. The RRs for Hispanics, Blacks, and other races were greater than those for Whites; the RRs across different age categories were all significantly positive.

**Conclusion:**

This study confirms an association between long-term NO_3_ exposure in drinking water and the incidence of colorectal cancer in California, emphasizing the need for stringent water quality control and public health strategies to address this risk, particularly in vulnerable populations.

## Introduction

California is the United States’ leading state in agricultural production, generating nearly 400 distinct commodities, including more than 30% of the nation’s vegetables and 60% of its fruits and nuts. This extensive agricultural activity has led to substantial use of nitrogen fertilizers, contributing to significant nitrate (NO_3_) leaching into the state's water resources [1–3]. Notably, California reports the highest average NO_3_ concentrations exceeding the maximum contaminant level (MCL) in drinking water compared to any other state, with a considerable number of people served by systems that violate NO_3_ standards [3].

The current MCL for NO_3_ in public drinking water systems is set at 45 mg/l as NO_3_ or 10 mg/l as N, primarily aiming to protect infants from methemoglobinemia [4]. However, this standard does not address the risks associated with other potential health issues stemming from long-term NO_3_ exposure. Extensive research indicates that chronic exposure to elevated NO_3_ levels is linked to various health risks, including cancers such as colorectal cancer, mediated by the endogenous formation of N-nitroso compounds in the gastrointestinal tract [2, 3, 5–8]. The cited papers provide a detailed explanation of carcinogenic N-nitroso compounds and the mechanisms of their formation within the human body [6, 7, 9–14].

Despite the established risks, studies examining the relationship between NO_3_ levels in drinking water and the risk of developing colorectal cancer have yielded inconsistent findings [15]. These studies vary in design, including ecological, case–control, and cohort studies, with the latter two linking exposure–response at the individual level. Such studies consider individual water consumption patterns, which can vary widely based on factors such as age, sex, ethnicity, socioeconomic status, and seasonal changes [16].

This study hypothesizes that an increase in the concentration of NO_3_ in California’s public drinking water systems is positively associated with the incidence rate of colorectal cancer. To test this hypothesis, we conducted a time-series analysis examining the association between county-level changes in average NO_3_ concentration in drinking water sources from 1990 to 2015 and diagnoses of colorectal cancer from 2010 to 2015 in California, USA, using a generalized linear model of the Poisson family.

## Material and methods

### Data and participants

Data on colon and rectum cancer incidence for the years 2010 to 2015 were acquired from the Surveillance, Epidemiology, and End Results (SEER) program in California, USA. The diagnoses of colorectal cancer were identified utilizing the International Classification of Diseases for Oncology, Third Edition (ICD-O-3) codes C180, C182-C189, C199, C209, which include primary tumor sites such as the cecum (C180), ascending colon (C182), hepatic flexure of the colon (C183), transverse colon (C184), splenic flexure of the colon (C185), descending colon (C186), and sigmoid colon (C187). The SEER program, which covers approximately 34.6 percent of the U.S. population, compiles comprehensive cancer incidence data from population-based cancer registries (SEER, 2018). This dataset includes detailed patient demographics, primary tumor site, tumor morphology, stage at diagnosis, and initial treatment modalities, and also provides follow-up on patient vital status. In the present study, a count variable representing the number of diagnoses of colorectal cancer within each county was generated to serve as the group variable. Only patients whose initial diagnosis corresponded to one of the specified ICD-O-3 codes were included in the analysis.

For this study, patient selection was strictly confined to individuals diagnosed with primary colorectal cancer, utilizing the SEER program’s ‘Sequence Number’ variable. This variable was employed to identify and include only those patients whose ‘Sequence Number’ was recorded as ‘00’, indicating the presence of a single primary tumor (i. e. One primary only in the patient’s lifetime). This methodological choice allowed us to rigorously exclude any patients with multiple primary cancers or those whose cancer had metastasized, thereby ensuring that our analysis accurately focused on the association between NO_3_ exposure and the incidence of primary colorectal cancer alone.

### Study design

This study employs a time-series analysis using a generalized linear model of the Poisson family to investigate the association between exposure to increased concentrations of NO_3_ in drinking water and the incidence of colon and rectum cancer in California from 1990 to 2015. The dependent variable is the monthly count of colorectal cancer diagnoses in each county from 2010 to 2015. The independent variables include NO_3_, disinfection byproducts (DBPs), and N-nitroso compounds (NDMA and NDEA), with data collected from various sampling stations within each county over the study period. An average concentration from all stations was computed to represent the exposure level for the population of each county. To address overdispersion—where the variance of the outcome counts exceeds that predicted by a Poisson distribution—a Pearson chi-square statistic divided by the residual degrees of freedom was used as a scale parameter [17].

### Exposure assessment

Water quality data were obtained from the California State Water Resources Control Board via the Electronic Data Transfer (EDT) Library and the Water Quality Analyses Data and Download Page [18]. This comprehensive dataset encompasses nearly 580 different water quality parameters, from which concentrations of NO_3_ and the sum of DBPs were specifically extracted. The sum of DBPs included trihalomethanes (THMs) such as chloroform, bromodichloromethane, chlorodibromomethane, and bromoform, along with haloacetic acids (HAA5), comprising monochloroacetic acid, dichloroacetic acid (DCA), trichloroacetic acid (TCA), monobromoacetic acid, and dibromoacetic acid. Additionally, data on N-nitroso compounds, specifically N-nitrosodiethylamine (NDEA) and N-nitrosodimethylamine (NDMA), were extracted for analysis.

The dataset includes 19 different water sampling sources. For this study, samples were selectively extracted from active treated sources (water sampled post-treatment), treated water within the distribution system (sample points post-treatment within the system), combined treated sources (combined sources that are treated), and purchased water (purchased source water sampled post-treatment). These samples were chosen to focus exclusively on water intended for drinking purposes, representing points where the water consumed by county residents is most likely sourced. For the N-nitroso compounds, sampling points included entry points to the distribution system (EPTDS) and points of maximum residence time within the distribution system (DSMRT), which are defined as locations where the water has resided in the system the longest relative to the EPTDS [19, 20].

The selection of sampling points is crucial for assessing water treatment efficacy and public health implications. Active Treated (AT) points evaluate treatment effectiveness at active sources, ensuring compliance with quality standards. Combined Treated (CT) points handle water from multiple sources, assessing the ability of treatment processes to maintain consistent quality. Distribution system Treated (DT) points reveal potential quality changes or recontamination risks in the distribution infrastructure, which are critical for understanding water quality at consumer taps. Purchased Treated (PT) points assess externally sourced water, ensuring it meets local safety standards before integration. This focus on treated water sampling provides vital data for enhancing water treatment and safety, aligning with public health goals and offering a robust framework for improving drinking water management.

Data on NO_3_ and DBPs from 1990 to 2015 were available for 25 counties in California: Alameda, Butte, Calaveras, Contra Costa, Fresno, Kern, Kings, Lake, Los Angeles, Marin, Nevada, Placer, Riverside, Sacramento, San Bernardino, San Diego, San Joaquin, San Luis Obispo, San Mateo, Santa Barbara, Santa Clara, Santa Cruz, Solano, Stanislaus, and Ventura. Given that nearly 30 million of California’s 37 million residents reside within these counties, the study was concentrated in these areas. The limited availability of data on N-nitroso compounds, particularly outside Los Angeles County, precluded their inclusion in the regression analysis across all counties. Consequently, the exploration of the correlation between increased concentrations of N-nitroso compounds and colorectal cancer diagnosis was confined to Los Angeles County. Within each county, multiple sampling stations provided data; therefore, an average value from all stations was computed to represent the NO_3_ concentration in the drinking water for each county.

This study primarily focused on DBPs resulting from chlorination, such as trihalomethanes and halo acetic acids, due to the prevalent use of chlorine-based methods in the sampled regions and the comprehensive data available for these compounds. While acknowledging that other disinfectants like ozone also produce DBPs, the lack of detailed data on these methods limited our analysis. Future research should explore the effects of various disinfection practices to fully assess their health impacts.

### Statistical analysis

In this study, a generalized linear model of the Poisson family was applied to estimate the association between an increase in NO_3_ and an increase in the count of colorectal cancer. The model used is as follows:

$${Y}_{t} \sim Poisson \left({\mu }_{t}\right)$$

$$\text{log}\left({\mu }_{t}\right)= \alpha +{\beta }_{1}\left({NO}_{3}\right)+ {\beta }_{2}\left(DBPs\right)+{\beta }_{3}\left(NOC\right)+ f\left({Time}_{t}, 4\right)+Calendar Time,$$

where *t* is the month of the observation, *Y*_*t*_ is the count of colorectal cancer on month t, α is the intercept, month is the date of each month patients were diagnosed with colorectal cancer, *f (Time*_*t*_*, 4)* is Fourier series terms and coefficients as a linear term to capture long-term trend; the coefficients of the sin/cos terms are estimated by maximum likelihood such that the linear combination models the seasonal patterns in the outcome data as closely as possible. To control for potential confounders such as sex, age at diagnosis, and race/ethnicity our analysis was stratified by these variables. This approach was chosen to mitigate the risk of ecological fallacy, which arises from making inferences about individual characteristics from aggregated data. Direct comparisons between these stratified groups were deliberately avoided because our dataset lacks individual-level data. Instead, the analysis was conducted separately within each stratum to ensure that confounding factors were appropriately accounted for, while still providing a robust assessment of the associations observed.

To account for seasonal and long-term patterns, periodic functions (Fourier series terms) were employed in this study. These functions, comprising pairs of sine and cosine functions, are adept at capturing regular seasonal variations [21]. The model included four harmonics (four sine/cosine pairs) to delineate seasonality, supplemented by a linear time function to capture broader temporal trends. Data merging involved the monthly colorectal cancer diagnosis counts for each county from 2010 to 2015, with the mean concentration of NO_3_ measured in mg/L as NO_3_ for the preceding one-year (lag 0–1), five-year (lag 0–5), ten-year (lag 0–10), fifteen-year (lag 0–15), and twenty-year (lag 0–20) periods. DBPs were incorporated as confounders, and potential modifiers such as sex, age at diagnosis, and race/ethnicity were examined. Since individual-level data were not utilized in this analysis, direct comparisons between groups (sex, race, age, and county of residence) were not conducted to avoid ecological fallacy. The reported RR and 95% confidence intervals (CI) are based on each 1 mg/L increase in NO_3_ concentration. All statistical analyses were conducted using STATA V. 14 (College Station, TX).

## Results

### The descriptive analysis of the SEER data

In this study, the demographic and clinical characteristics of patients diagnosed with colorectal cancer were examined (Table 1). The gender distribution of the studied population was nearly equal, with females comprising 48% and males 52%. The racial composition included Non-Hispanic Whites (53%), Non-Hispanic Blacks (8%), Hispanics (22%), and other races (16%). The age distribution showed that 4% of the patients were diagnosed under the age of 40, 43% between 41 and 64 years, 49% between 65 and 90 years, and 4% were older than 90. A notable increase in colorectal cancer diagnoses among individuals under 40 years was observed, rising from 383 cases in 2000 to 610 in 2015 (Appendix Table 5). Histologically, 86.41% of the cases were classified as ‘Adenocarcinoma’ and 8.18% as ‘cystic, mucinous, and serous neoplasms.’ Of all colorectal cancer cases diagnosed from 2010 to 2015, 97.89% showed positive histology (tissue microscopically examined), while 0.27% were confirmed by positive cytology (fluid cells microscopically examined without tissue).

**Table 1:** Characteristics of Colorectal Cancer diagnosis during 2010 to 2015 within the study area in California, USA (n = 56,631)

Characteristics	#Colorectal cancer	Percent (%)
Sex		
Female	27,842	47.9
Male	30,170	52.0
Race		
White (Non-hispanic)	30,969	53.4
Black (Non-hispanic)	4,831	8.33
Hispanic	12.923	22.3
Others	9,289	16.0
Age		
< = 40	2455	4.2
41–64	24,999	43.1
65–90	28,776	49.6
> = 91	1,782	3.1
^*^Primary site of cancer		
C180-Cecum	8,382	14.8
C182-Ascending colon	7,145	12.6
C183-Hepatic flexure of colon	1,814	3.2
C184-Transverse colon	3,455	6.1
C185-Splenic flexure of colon	1,098	1.9
C186-Descending colon	2,298	4.1
C187-Sigmoid colon	11,710	20.6
C188-Overlapping lesion of colon	499	0.8
C189-Colon, NOS	2,093	3.7
C199-Rectosigmoid junction	4,386	7.7
C209-Rectum, NOS	13,718	24.2
Total population	56,631	100.0

^*The primary sites are based on ICD^^−^^9 code^

In this study, the characteristics of patients diagnosed with colorectal cancer were analyzed on a county-by-county basis to elucidate the demographic distribution of cases across sex, race, age, total population, and colorectal cancer incidence per 100,000 persons (Table 2). The 25 counties included in this analysis were selected based on the availability of NO_3_ and DBP data and represent a combined population of nearly 30 million, out of the total 37 million residents in California as of 2010. The analysis revealed that Los Angeles County recorded the highest number of colorectal cancer diagnoses with 18,711 individuals (unadjusted count), whereas Calaveras County had the lowest with 121 individuals (unadjusted count). This granular analysis provides a deeper understanding of the regional variations in colorectal cancer incidence, aiding in targeted public health responses and resource allocation.

**Table 2:** Characteristics of colorectal cancer incidence during 2010 to 2015 separated by county in California, USA

Characteristics	Sex (%)		Race (%)				Age of Diagnosis (%)				Colorectal cancer count (%)
	Female	Male	Whites	Blacks	Hispanics	Other races	> = 40	41–64	65–90	> = 91	
Alameda	1372 (48.1)	1479 (51.8)	1204 (42.2)	460 (16.1)	360 (12.6)	827 (29.1)	123 (4.3)	1297 (45.4)	1329 (46.6)	102 (3.5)	2851 (100.0)
Butte	275 (51.6)	257 (48.3)	466 (87.5)	4 (0.7)	32 (6.1)	30 (5.6)	15 (2.8)	197 (37.1)	301 (56.5)	19 (3.5)	532 (100.0)
Calaveras	47 (38.8)	74 (61.1)	109 (90.1)	1 (0.8)	8 (6.6)	3 (2.4)	2 (1.6)	44 (36.3)	66 (54.5)	9 (7.4)	121 (100.0)
Contra Costa	1146 (49.1)	1192 (50.9)	1453 (62.1)	246 (10.5)	299 (12.7)	340 (14.5)	103 (4.4)	1066 (45.5)	1093 (46.7)	76 (3.2)	2338 (100.0)
Fresno	736 (47.6)	809 (52.3)	753 (48.7)	92 (5.9)	529 (34.2)	171 (11.1)	78 (5.1)	645 (41.7)	770 (49.8)	52 (3.3)	1545 (100.0)
Kern	606 (44.4)	758 (55.5)	820 (60.1)	79 (5.7)	375 (27.4)	90 (6.6)	81 (5.9)	626 (45.8)	629 (46.1)	28 (2.1)	1364 (100.0)
Kings	96 (44.4)	120 (55.5)	104 (48.1)	12 (5.6)	86 (39.8)	14 (6.4)	13 (6.1)	96 (44.4)	100 (46.3)	7 (3.2)	216 (100.0)
Lake	97 (51.3)	92 (48.6)	161 (85.1)	4 (2.1)	13 (6.8)	11 (5.8)	2 (1.1)	79 (41.8)	102 (53.9)	6 (3.2)	189 (100.0)
Los Angeles	9074 (48.5)	9637 (51.5)	7643 (40.8)	2106 (11.2)	5280 (28.2)	3682 (19.6)	735 (3.9)	8076 (43.1)	9329 (49.8)	571 (3.1)	18,711 (100.0)
Marin	290 (50.9)	279 (49.1)	490 (86.1)	11 (1.9)	31 (5.4)	37 (6.5)	8 (1.4)	242 (42.5)	299 (52.5)	20 (3.5)	569 (100.0)
Nevada	100 (42.9)	133 (57.1)	213 (91.4)	1 (0.4)	13 (5.6)	6 (2.6)	12 (5.1)	89 (38.2)	120 (51.5)	12 (5.1)	233 (100.0)
Placer	375 (47.8)	409 (52.1)	658 (83.9)	9 (1.1)	60 (7.6)	57 (7.3)	26 (3.3)	296 (37.7)	437 (55.7)	25 (3.2)	784 (100.0)
Riverside	2070 (46.8)	2352 (53.2)	2701 (61.1)	304 (6.9)	1080 (24.4)	337 (7.6)	165 (3.7)	1757 (39.7)	2389 (54.1)	111 (2.5)	4422 (100.0)
Sacramento	1482 (49.4)	1515 (50.5)	1839 (61.3)	310 (10.3)	354 (11.8)	494 (16.4)	122 (4.1)	1283 (42.8)	1512 (50.4)	80 (2.7)	2997 (100.0)
San Bernardino	1758 (45.2)	2133 (54.8)	1975 (50.7)	401 (10.3)	1213 (31.2)	302 (7.7)	160 (4.1)	1746 (44.8)	1902 (48.8)	83 (2.1)	3891 (100.0)
San Diego	2799 (48.5)	2975 (51.5)	3660 (63.4)	271 (4.7)	1106 (19.1)	737 (12.7)	289 (5.1)	2394 (41.5)	2884 (50.0)	207 (3.6)	5774 (100.0)
San Joaquin	641 (48.0)	696 (52.1)	687 (51.4)	130 (9.7)	299 (22.4)	221 (16.5)	58 (4.3)	588 (44.0)	657 (49.1)	34 (2.5)	1337 (100.0)
Obispo	306 (50.5)	300 (49.5)	506 (83.5)	9 (1.5)	60 (9.9)	31 (5.1)	22 (3.6)	226 (37.2)	338 (55.8)	20 (3.3)	606 (100.0)
San Mateo	701 (48.5)	745 (51.5)	809 (55.6)	43 (2.9)	202 (13.9)	392 (27.1)	60 (4.1)	629 (43.5)	702 (48.5)	55 (3.8)	1446 (100.0)
Santa Barbara	382 (48.97)	398 (51.1)	505 (64.7)	20 (2.5)	201 (25.7)	54 (6.9)	33 (4.2)	314 (40.2)	394 (50.5)	39 (5.0)	780 (100.0)
Santa Clara	1600 (47.8)	1747 (52.2)	1672 (49.9)	99 (2.9)	519 (15.5)	1057 (31.5)	160 (4.7)	1567 (46.8)	1504 (44.9)	116 (3.4)	3347 (100.0)
Santa Cruz	217 (46.2)	252 (53.7)	338 (72.1)	6 (1.2)	99 (21.1)	26 (5.5)	19 (4.1)	224 (47.7)	216 (46.1)	10 (2.1)	469 (100.0)
Solano	425 (47.1)	477 (52.8)	479 (53.1)	149 (16.5)	103 (11.4)	171 (18.9)	45 (4.9)	421 (46.7)	418 (46.4)	18 (2.0)	902 (100.0)
Stanislaus	513 (48.7)	540 (51.2)	682 (64.7)	35 (3.3)	269 (25.5)	67 (6.4)	56 (5.3)	459 (43.6)	514 (48.8)	24 (2.3)	1053 (100.0)
Ventura	734 (47.8)	801 (52.2)	1042 (67.8)	29 (1.9)	332 (21.6)	132 (8.6)	68 (4.4)	638 (41.5)	771 (50.2)	58 (3.7)	1535 (100.0)

### Trend analysis of colorectal cancer counts from 2010 to 2015

**Fig. 1:**
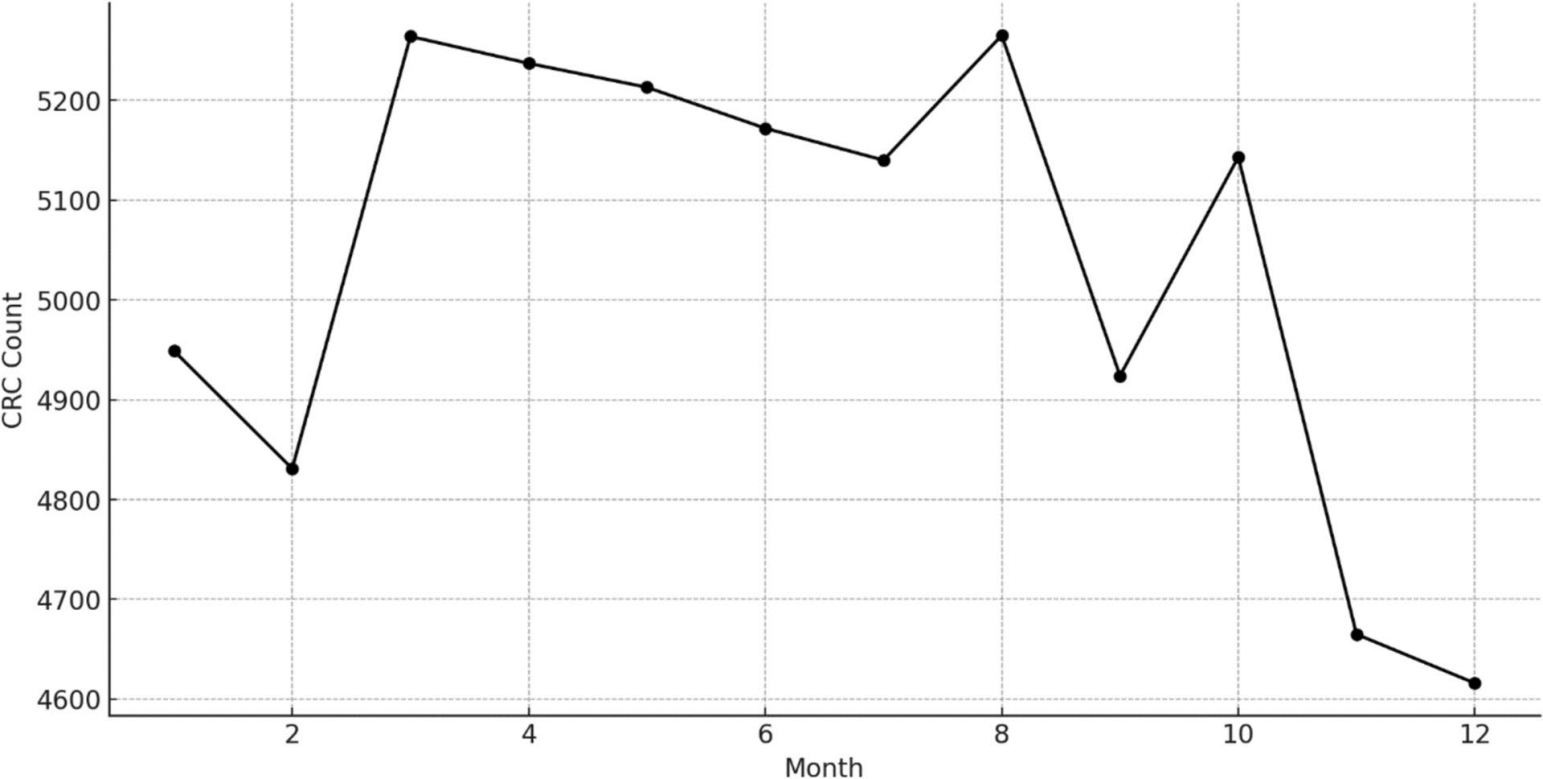
Trend of monthly counts of colorectal cancer cases from January 2010 to December 2015

The trend of colorectal cancer counts from January 2010 to December 2015 is depicted in Fig. 1. The data represents monthly counts of colorectal cancer cases, encompassing various ICD-10 codes related to colon and rectum cancer. These include C180 (Cecum), C182 (Ascending colon), C183 (Hepatic flexure of colon), C184 (Transverse colon), C185 (Splenic flexure of colon), C186 (Descending colon), and C187 (Sigmoid colon).

Overall, the trend exhibits noticeable fluctuations in the monthly counts of colorectal cancer cases over the six-year period. The counts show a general pattern of variability with peaks and troughs occurring at different times throughout the years. Notably, there are several months with elevated counts which may correspond to specific seasonal or external factors influencing the incidence of colorectal cancer. The trend does not indicate a consistent upward or downward trajectory, suggesting that while there are periods of higher incidence, these do not follow a straightforward temporal pattern. Our analysis of monthly cancer case counts reveals variations that are not fully explained by the data at hand. Although March is observed nationally as Colorectal Cancer Awareness Month, potentially increasing screenings like colonoscopies, our dataset lacks specific monthly screening data for California. Therefore, without comprehensive contextual information, we refrain from attributing observed trends to specific factors. This highlights a limitation of our study and underscores the need for future research to integrate detailed healthcare utilization data to better understand these patterns.

### The descriptive analysis of NO_*3*_ in drinking water

**Fig. 2:**
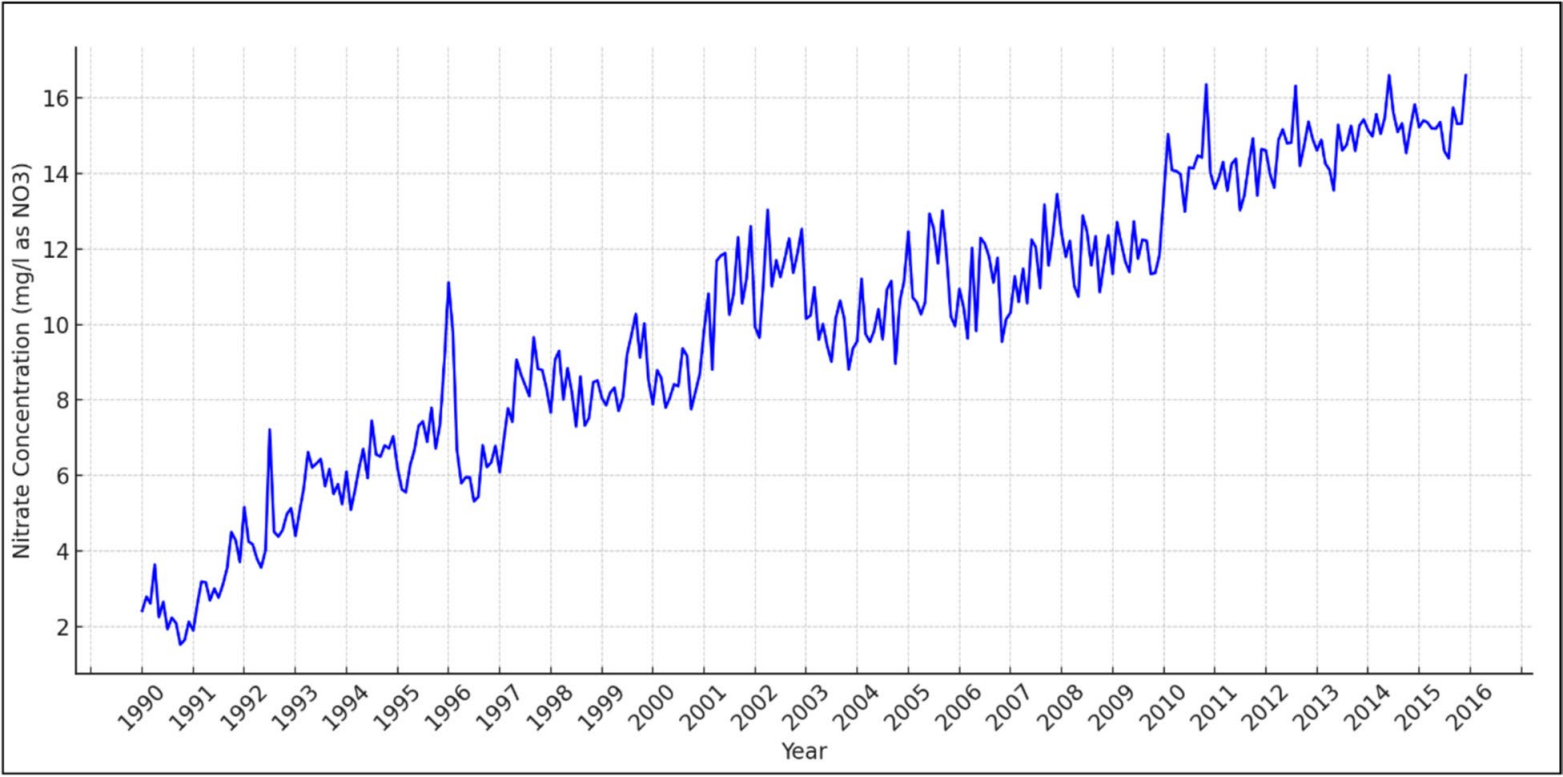
The temporal trend of monthly average concentration of nitrate (mg/l as NO_3_) in California, USA from 1990 to 2015. Some non−detects (ND) in the time series are reported as 0.000 instead of <0.5 (assuming 0.5 is the detection limit) in the FINDING field. This representation occurs when the laboratory elects to report data that it has detected below the State detection limit (DLR). If these reports derive from the same analyses, it indicates a decision to disclose detected values beneath the detection limit for transparency. In cases of different analyses, the use of either <DLR or ND in the findings is simply a preference of data entry; both terms essentially imply the same result—below detection limit findings

The monthly average concentration of NO_3_ in drinking water has increased steadily from 1990 to 2015 in California, USA, as can be seen from Fig. 2. The observed spikes in NO_3_ levels in California drinking water in 1995 and 2010 are likely linked to increased nitrogen fertilizer use, variable weather conditions affecting NO_3_ leaching into groundwater, and shifts in water management practices. These factors collectively influence NO_3_ fluctuations, though specific causes for each spike would require further detailed analysis of local agricultural activities and environmental conditions during those periods [3].

In this study, an analysis of the monthly average concentrations of DBPs in drinking water across California, USA from 1990 to 2015 revealed a significant increase, with a notable surge observed from 2009 to 2010 (Appendix Figure 3). Additionally, the concentration of N-nitroso compounds was evaluated during the same period (Appendix Figure 4), showing a continuous increase from 1990 to 2015, despite the maximum contaminant level (MCL) for these compounds being set at 0 µg/l. It is important to note that approximately 90% of the N-nitroso compound data were sourced from Los Angeles County, with the remaining 10% distributed among the other 24 counties.

Further investigation was conducted into the population served by either surface or groundwater sources and the trends in NO_3_ violations in drinking water across the selected counties (Table 3). The findings indicate that the highest number of NO_3_ violations occurred in San Bernardino (1021 instances), Los Angeles (945 instances), and Fresno (729 instances), with most violations emerging after the year 2000. Specifically, 46.41% of the violations occurred between 2010 and 2015, 33.00% between 2005 and 2009, and 14.79% between 2000 and 2004. The counties of Los Angeles, San Diego, San Bernardino, and Fresno served the largest populations through systems that frequently violated NO_3_ standards, highlighting critical areas for targeted regulatory oversight and public health interventions.

**Table 3:** The public water system characteristics in the study area: trends in drinking water NO_3_ violations across the study area

County	Connections to PWSs	Population served by surface water	Population served by groundwater	Total population served	# violation of the standard level of 45 mg/l-NO_3_ or 10 mg/l-N by each drinking water by the type of sampling station					Percent (%) of total violation in prior years (separated five years from 2015 to 1990)				
					AT	CT	DT	PT	Total	2015–2010	2009–2005	2004–2000	1999–1995	1994–1990
Alameda	480,993	1,379,040	396,700	1,775,740	7	0	0	1	8	25.00	50.00	12.50	12.50	0.00
Butte	45,994	41,600	110,698	152,298	2	0	0	0	2	0.00	50.00	0.00	0.00	50.00
Calaveras	8197	4016	21,397	25,413	0	0	0	0	0	0.00	0.00	0.00	0.00	0.00
Contra Costa	127,542	354,148	68,700	422,848	16	0	0	0	16	43.75	43.75	12.50	0.00	0.00
Fresno	183,556	647,763	83,452	731,215	729	0	0	0	729	48.01	30.45	5.49	16.05	0.00
Kern	145,523	316,984	218,076	535,060	60	87	0	0	147	47.62	27.21	25.17	0.00	0.00
Kings	4960	12,000	24,813	36,813	0	0	0	0	0	0.00	0.00	0.00	0.00	0.00
Lake	3007	6499	100	6599	0	0	0	0	0	0.00	0.00	0.00	0.00	0.00
Los Angeles	1,221,536	6,409,402	562,769	6,972,171	662	281	2	0	945	63.70	18.73	1.80	13.97	1.80
Marin	82,627	251,110	1700	252,810	0	0	0	0	0	0.00	0.00	0.00	0.00	0.00
Nevada	15,673	45,454	0	45,454	0	0	0	0	0	0.00	0.00	0.00	0.00	0.00
Placer	52,848	166,797	30	166,827	1	0	0	0	1	100.00	0.00	0.00	0.00	0.00
Riverside	327,754	1,049,770	220,765	1,270,535	68	46	0	1	115	21.74	39.13	38.26	0.00	0.87
Sacramento	246,240	788,483	13,386	801,869	0	0	0	0	0	0.00	0.00	0.00	0.00	0.00
San Bernardino	382,221	1,005,466	472,454	1,477,920	50	965	1	5	1021	33.89	37.51	27.72	0.88	0.00
San Diego	351,836	1,710,828	16,270	1,727,098	170	0	3	0	173	84.39	13.87	1.73	0.00	0.00
San Joaquin	116,171	237,039	254,163	491,202	2	0	0	0	2	50.00	50.00	0.00	0.00	0.00
San Luis Obispo	35,582	14,415	82,125	96,540	60	0	0	0	60	31.67	61.67	0.00	5.00	1.67
San Mateo	39,950	168,736	0	168,736	18	1	0	2	21	0.00	90.48	9.52	0.00	0.00
Santa Barbara	65,112	254,798	80	254,878	9	0	0	0	9	55.56	44.44	0.00	0.00	0.00
Santa Clara	11,169	0	42,262	42,262	79	7	0	0	86	68.60	29.07	2.33	0.00	0.00
Santa Cruz	42,208	96,142	48,229	144,371	0	0	0	0	0	0.00	0.00	0.00	0.00	0.00
Solano	90,236	315,352	1455	316,807	4	0	0	0	4	100.00	0.00	0.00	0.00	0.00
Stanislaus	11,601	865	47,902	48,767	125	78	0	0	123	4.88	85.37	72.36	2.44	0.00
Ventura	96,306	250,962	142,677	393,639	70	7	0	3	80	1.25	93.75	5.00	0.00	0.00
Total	4,188,842	15,527,669	2,830,203	18,357,872	2132	1472	6	12	3542	46.41	33.00	14.79	7.48	0.56

*PWSs*: Public water systems; *AT*: an active source which is sampled after any treatment; *CT*: Combined sources which are treated; *DT*: Sample point within the distribution system after treatment; *PT*: Purchased source water which is sampled after any treatment

### The adjusted model: a generalized linear analysis

The association between the increase in the concentration of NO_3_ in drinking water and colorectal cancer in California, USA was investigated in this study. In the unadjusted model (Appendix Table 7), the association between the increase in concentration of NO_3_ in drinking water and colorectal cancer in California, USA was found positively significant. Based on the results, 1 mg/l increase in the concentration (as NO_3_) of NO_3_ at lag 0–1, lag 0–5, lag 0–10, lag 0–15 and lag 0–20 is associated with colorectal cancer RR of 5.1% [RR: 1.051 (95% CI: 1.049, 1.055)], 6% [RR: 1.060 (95% CI: 1.058, 1.061)], 5.1% [RR: 1.051 (95% CI: 1.049, 1.053)], 4.4% [RR: 1.044 (95% CI: 1.043, 1.046)], and 6% [RR: 1.060 (95% CI: 1.059, 1.061)], respectively. The association between the increase in the concentration of DBPs and increase in the number of colorectal cancer patients was also investigated separately (Appendix Table 8). The results showed that there is no association between the increase in DBPs and colorectal cancer increase in the number of colorectal cancer patients. The cumulative NO_3_ exposure and colorectal cancer diagnosis, while controlling for DBPs, was investigated, as shown in Table 4. The results showed that the increase in the concentration of NO_3_ during the past 5 years has a higher RR, compared with other lags. It was found that 1 mg/l increase in the concentration (as NO_3_) of NO_3_ at lag 0–5, while controlling for DBPs, is associated with colorectal cancer RR of 6.6% [RR: 1.066 (95% CI: 1.063, 1.069)].

**Table 4:** The association between cumulative NO_3_ (mg/l as NO_3_) exposure and colorectal cancer diagnosis during 2010 to 2015 in California, while controlling for DBPs

Characteristics	Lag 0–1	Lag 0–5	Lag 0–10	Lag 0–15	Lag 0–20
	RRs (95% CI)	RRs (95% CI)	RRs (95% CI)	RRs (95% CI)	RRs (95% CI)
Total	1.056 (1.055, 1.058)	1.066 (1.063, 1.069)	1.030 (1.028, 1.031)	1.017 (1.016, 1.018)	1.035 (1.034, 1.037)
Sex					
Female	1.056 (1.054, 1.058)	1.066 (1.061, 1.070)	1.029 (1.027, 1.032)	1.017 (1.015, 1.019)	1.036 (1.034, 1.038)
Male	1.055 (1.053, 1.060)	1.062 (1.059, 1.066)	1.029 (1.027, 1.031)	1.017 (1.015, 1.018)	1.035 (1.033, 1.037)
Race					
White	1.070 (0.728, 1.574)	1.033 (1.021, 1.045)	1.001 (0.997, 1.003)	0.988 (0.985, 0.991)	1.008 (1.005, 1.011)
Black	1.052 (1.047, 1.058)	1.076 (1.046, 1.108)	1.038 (1.030, 1.045)	1.019 (1.014, 1.024)	1.033 (1.028, 1.038)
Hispanic	1.034 (1.030, 1.037)	1.061 (1.059, 1.063)	1.033 (1.031, 1.035)	1.023 (1.021, 1.024)	1.037 (1.035, 1.039)
Others	1.058 (1.055, 1.061)	1.078 (1.072, 1.084)	1.052 (1.048, 1.057)	1.038 (1.034, 1.041)	1.057 (1.053, 1.060)
Age					
< = 40	1.040 (1.034, 1.046)	1.037 (1.030, 1.043)	1.015 (1.009, 1.022)	1.006 (1.000, 1.012)	1.022 (1.016, 1.028)
41–64	1.055 (1.053, 1.057)	1.063 (1.059, 1.067)	1.030 (1.028, 1.032)	1.017 (1.015, 1.019)	1.036 (1.034, 1.038)
65–90	1.056 (1.054, 1.058)	1.065 (1.060, 1.069)	1.028 (1.026, 1.031)	1.017 (1.015, 1.019)	1.036 (1.034, 1.038)
> 90	1.048 (0.883, 1.245)	1.046 (1.038, 1.055)	1.027 (1.018, 1.035)	1.017 (1.010, 1.024)	1.031 (1.024, 1.039)

*DBPs*: disinfection by products: sum of THMs (chloroform, bromodichloromethane, chlorodibromomethane and bromoform) and HAA5 (monochloroacetic acid, dichloroacetic acid (DCA), trichloroacetic acid (TCA), monobromoacetic acid, and dibromoacetic acid), *RR*: Risk Ratio, *CI*: Confidence Interval

Hypothesizing that patients’ characteristics who diagnosed with colorectal cancer could modify the effect of NO_3_ on the increase in the colorectal cancer diagnosis, the effect of sex, race and age was investigated in this study (Table 4). The results showed that the highest RR for both Males and Females is at lag 0–5. It was found that 1 mg/l increase in the concentration (as NO_3_) of NO_3_ at lag 0–5, while controlling for DBPs, is associated with colorectal cancer RR of 6.6% [RR: 1.066 (95% CI: 1.061, 1.070)] and 6.2% [RR: 1.062 (95% CI: 1.059, 1.066)] among Females and Males, respectively. In addition, it was found that 1 mg/l increase in the concentration (as NO_3_) of NO_3_ at lag 0–5 is associated with colorectal cancer RR of 6.1% [RR: 1.061 (95% CI 1.059, 1.063)], 7.6% [RR: 1.076 (95% CI: 1.048, 1.101)], 3.3% [RR: 1.033 (95% CI: 1.021, 1.045)], and 7.8% [RR: 1.078 (95% CI: 1.072, 1.084)] among Hispanics, Blacks, Whites and Other races, respectively. Furthermore, 1 mg/l increase in the concentration (as NO_3_) of NO_3_ at lag 0–5 was found to be associated with colorectal cancer RR of 3.7% [RR: 1.037 (95% CI: 1.030, 1.043)], 6.3% [RR: 1.063 (95% CI: 1.059, 1.067)], 6.5% [RR: 1.065 (95% CI: 1.060, 1.069)], and 4.6% [RR: 1.046 (95% CI: 1.038, 1.055)] among under 40, 41 to 64, 65 to 90 and older than 90 years old patients diagnosed with colorectal cancer, respectively.

## Discussion

This study represents the first time-series analysis conducted in California aimed at investigating the association between long-term exposure to NO_3_ in drinking water and the incidence of colorectal cancer. The primary aim of this research is to explore the potential link between NO_3_ concentrations in California’s public drinking water and colorectal cancer incidence rates. Recognizing the inherent limitations of time-series analysis compared to more precise cohort studies, this research serves as a foundational step toward more comprehensive investigations. Given the elevated levels of NO_3_ found in California’s drinking water and its association with various diseases, including colorectal cancer, there is a pressing need for initial studies that can guide and inform more detailed, subsequent research. This study seeks to provide initial insights and generate data that will underpin the development of targeted cohort studies, thereby addressing a critical public health issue in the state of California.

The findings reveal a consistently positive association across all examined lags between NO_3_ exposure and colorectal cancer incidence, affirming the hypothesis that increasing concentrations of NO_3_ in public drinking water systems correlate with rising rates of colorectal cancer. However, stratification by race/ethnicity and age at diagnosis revealed significant variations in these associations.

Several strengths bolster this study. It utilized NO_3_ data collected from California’s public drinking water systems over a 25-year period, enabling comprehensive exposure assessments. The incorporation of five latency periods, covering up to two decades of exposure prior to diagnosis, was critical for capturing long-term effects. The use of cancer incidence rates, rather than mortality data, strengthens the findings, as incidence is more closely linked to cancer etiology [22]. Moreover, the high-quality SEER cancer registry data allowed for the control of confounders such as DBPs and N-nitroso compounds, further validating the results.

These findings are supported by a growing body of literature. For instance, Schullehner et al. [15] conducted a nationwide cohort study in Denmark and reported a similar positive association between NO_3_ exposure and colorectal cancer risk, particularly in cases of long-term exposure. This corroborates our findings and underscores the global relevance of NO_3_ contamination in drinking water as a public health issue. Similarly, De Roos et al. [23] found a comparable association in the United States, where long-term exposure to NO_3_ in public water supplies was linked to an increased risk of colon cancer.

The stratified analysis revealed significant disparities in nitrate-related colorectal cancer risks across racial and ethnic groups. Higher risk ratios (RRs) were observed among Hispanic, Black, and other minority populations compared to Whites. This pattern echoes findings from other studies, such as those by Pennino et al. [3], which documented higher NO_3_ levels in community water systems serving minority populations. Socioeconomic factors, dietary differences, and disparities in access to healthcare are likely contributors to these variations [23].

Interestingly, sex was not a significant modifier of colorectal cancer risk in this study, aligning with previous findings by Gulis et al., [22]. However, age did modify the effect of NO_3_ exposure, with higher RRs observed among younger age groups. This suggests that younger populations may be more vulnerable to the carcinogenic effects of NO_3_ exposure, possibly due to differences in diet, water consumption, or metabolic factors.

Several international studies have explored the association between NO_3_ in drinking water and various cancers, although the results have been mixed. Richards et al. [24] conducted an exposure assessment in New Zealand and estimated a significant health burden of colorectal cancer attributable to NO_3_ contamination. Similarly, a review by Ward et al. [8] underscored the carcinogenic potential of NO_3_, particularly through their conversion to N-nitroso compounds in the gastrointestinal tract. However, the exact mechanism by which NO_3_ increase cancer risk remains a subject of ongoing investigation.

Another important aspect of nitrate-related cancer risk is the potential interaction with other dietary factors. DellaValle et al. [25] examined the interaction between NO_3_ intake and dietary nitrites in a cohort of women in Shanghai and found that high NO_3_ consumption combined with high red meat intake significantly increased the risk of colorectal cancer. This interaction likely results from the formation of carcinogenic N-nitroso compounds in the presence of NO_3_ and nitrite, a hypothesis that is supported by experimental studies [26].

Moreover, Chang et al., [27] explored the role of water hardness (calcium and magnesium levels) in modifying the association between NO_3_ and rectal cancer risk. Their findings suggest that high calcium intake may mitigate the carcinogenic effects of NO_3_, although the evidence remains inconclusive. Similarly, Chiu et al., [28] examined the interaction between calcium and NO_3_ in drinking water, concluding that high calcium levels may lower the risk of colon cancer in populations exposed to high NO_3_ concentrations.

Essien et al. [29] conducted a systematic review and meta-analysis of studies investigating the relationship between NO_3_ exposure and cancer risk. Their meta-regression revealed significant associations between NO_3_ levels and colon cancer, aligning with the results of the current study. This meta-analysis reinforces the argument that NO_3_ contamination is a global concern, particularly for gastrointestinal cancers, and underscores the need for stricter regulation of NO_3_ levels in public water supplies.

The study’s limitations must also be considered. Although NO_3_ levels were rigorously measured, it was not feasible to adjust for all potential confounders, such as dietary habits, smoking, and medication use, which could influence colorectal cancer risk. Additionally, individual water consumption patterns vary, and the use of county-level NO_3_ concentrations may not fully capture individual exposure levels. Further research, incorporating individual-level exposure data, is necessary to refine these associations.

In conclusion, this study establishes a significant association between long-term exposure to NO_3_ in drinking water and the incidence of colorectal cancer in California, with particularly elevated risks among younger populations and racial minorities. These findings underscore the need for stringent water quality regulations and targeted public health interventions to mitigate the cancer risks associated with NO_3_ contamination.

## Figures and Tables

**Fig. 3:**
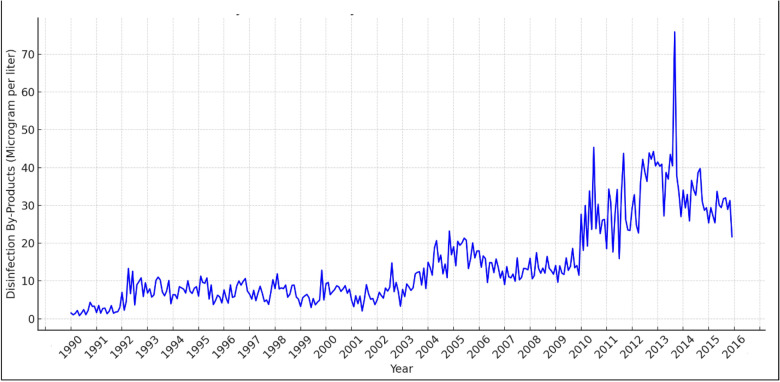
The temporal trend of the monthly average concentration of disinfection byproducts (DBPs) (µg/l) in California, USA from 1990 to 2015. Some non−detects (ND) in the time series are reported as 0.000 instead of <0.5 (assuming 0.5 is the detection limit) in the FINDING field. This representation occurs when the laboratory elects to report data that it has detected below the State detection limit (DLR). If these reports derive from the same analyses, it indicates a decision to disclose detected values beneath the detection limit for transparency. In cases of different analyses, the use of either <DLR or ND in the findings is simply a preference of data entry; both terms essentially imply the same result—below detection limit findings

**Fig. 4:**
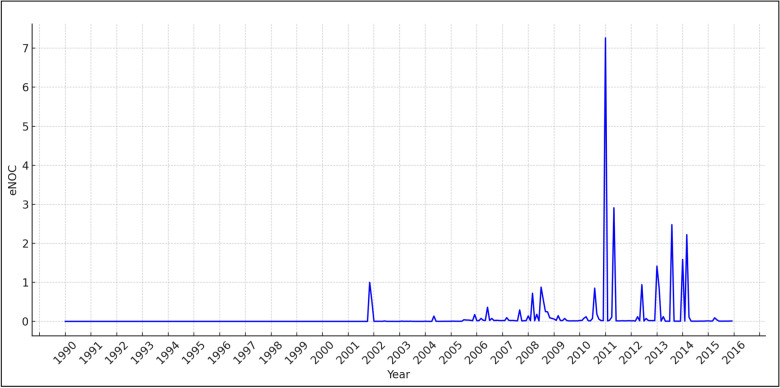
The temporal trend of the monthly average concentration of eNOC (µg/l) in Los Angeles, CA from 1998 to 2015. Some non−detects (ND) in the time series are reported as 0.000 instead of <0.5 (assuming 0.5 is the detection limit) in the FINDING field. This representation occurs when the laboratory elects to report data that it has detected below the State detection limit (DLR). If these reports derive from the same analyses, it indicates a decision to disclose detected values beneath the detection limit for transparency. In cases of different analyses, the use of either <DLR or ND in the findings is simply a preference of data entry; both terms essentially imply the same result—below detection limit findings

## Data Availability

The datasets generated during and/or analyzed during the current study are not publicly available due to [due to SEER policy in providing data] but are available from the corresponding author on reasonable request.

## Appendix

See Tables

**Table 5:** The incidence rate of colorectal cancer in USA from 2000 to 2015 among different age groups

Year	Age of the cases			
	< 40	40 to 64	65 to 90	> 90
2010	430	5108	6278	403
2011	465	5134	6048	357
2012	469	5053	6011	381
2013	492	5028	5861	362
2014	559	5374	5908	384
2015	619	5440	5774	394

^5^,

**Table 6:** The annual concentration of Nitrate (mg/l as NO_3_) in California, USA from 1990 to 2015

Year	Mean	SD	Minimum	Percentile			Maximum
				P25	P50	P75	
1990	2.33	4.07	0.00	0.40	1.00	3.00	46.90
1991	3.21	4.72	0.00	0.50	1.20	3.50	23.00
1992	4.65	7.76	0.00	0.50	1.40	5.75	89.00
1993	5.76	6.58	0.00	0.72	2.40	10.00	24.00
1994	6.39	7.59	0.00	0.54	2.20	10.70	34.67
1995	6.90	8.72	0.00	0.60	2.57	11.00	40.83
1996	6.85	9.16	0.00	0.50	2.00	10.00	45.00
1997	8.18	11.58	0.00	1.00	2.28	10.90	46.50
1998	8.24	11.37	0.00	1.20	2.42	12.16	47.00
1999	8.76	11.23	0.00	1.05	2.37	15.73	39.09
2000	8.43	11.45	0.00	1.00	2.23	14.19	55.00
2001	11.06	12.02	0.00	1.55	4.95	19.05	54.00
2002	11.46	11.00	0.00	2.00	6.28	19.25	47.00
2003	9.88	8.75	0.00	2.00	6.69	17.14	36.00
2004	10.23	8.23	0.00	2.44	8.18	17.05	28.77
2005	11.40	9.16	0.00	3.17	9.07	18.54	57.00
2006	10.97	8.63	0.00	2.33	10.09	18.36	35.00
2007	11.67	8.79	0.00	2.87	11.06	18.72	32.37
2008	11.86	8.68	0.00	3.46	11.68	18.10	39.50
2009	11.90	8.86	0.00	3.54	10.24	19.12	34.79
2010	15.10	6.96	0.00	11.20	16.30	17.62	75.00
2011	14.72	5.99	0.00	11.88	15.55	17.45	32.56
2012	15.77	6.76	0.00	12.89	17.03	18.69	45.90
2013	15.44	5.96	0.00	11.44	17.27	18.43	28.54
2014	16.48	6.91	0.00	10.83	18.40	19.42	36.11
2015	15.29	6.40	0.00	10.45	18.34	19.82	28.45
Total	14.80	7.17	0.00	9.94	16.46	19.15	89.00

^*SD*^^: S^^tandard Deviation^

6,

**Table 7:** The association between cumulative nitrate (mg/l as NO_3_) exposure and colorectal cancer diagnosis in California years 2010 to 2015

Characteristics	Lag 0–1	Lag 0–5	Lag 0–10	Lag 0–15	Lag 0–20
	RRs (95% CI)	RRs (95% CI)	RRs (95% CI)	RRs (95% CI)	RRs (95% CI)
Total	1.051 (1.049, 1.055)	1.060 (1.058, 1.061)	1.051 (1.049, 1.053)	1.044 (1.043, 1.046)	1.060 (1.059, 1.061)
*Sex*					
Female	1.056 (1.054, 1.058)	1.059 (1.057, 1.061)	1.050 (1.048, 1.053)	1.044 (1.042, 1.046)	1.060 (1.058, 1.062)
Male	1.055 (1.053, 1.058)	1.059 (1.056, 1.061)	1.050 (1.048, 1.052)	1.043 (1.041, 1.045)	1.059 (1.058, 1.0615)
*Race*					
White	1.059 (1.057, 1.060)	1.057 (1.055, 1.059)	1.044 (1.042, 1.046)	1.036 (1.034, 1.037)	1.046 (1.045, 1.048)
Black	1.052 (1.047, 1.058)	1.063 (1.058, 1.069)	1.055 (1.049, 1.060)	1.049 (1.044, 1.053)	1.064 (1.060, 1.068)
Hispanic	1.034 (1.030, 1.037)	1.032 (1.029, 1.036)	1.026 (1.022, 1.029)	1.023 (1.020, 1.026)	1.045 (1.042, 1.048)
Others	1.058 (1.055, 1.061)	1.075 (1.071, 1.079)	1.074 (1.070, 1.078)	1.067 (1.063, 1.070)	1.083 (1.080, 1.086)
*Age*					
< =40	1.040 (1.034, 1.046)	1.038 (1.032, 1.045)	1.029 (1.023, 1.036)	1.024 (1.018, 1.029)	1.036 (1.031, 1.041)
41–64	1.055 (1.053, 1.057)	1.059 (1.056, 1.061)	1.051 (1.048, 1.053)	1.044 (1.042, 1.046)	1.060 (1.058, 1.062)
65–90	1.056 (1.054, 1.058)	1.059 (1.057, 1.061)	1.050 (1.048, 1.052)	1.044 (1.042, 1.046)	1.060 (1.058, 1.062)
> 90	1.042 (1.035, 1.049)	1.046 (1.038, 1.054)	1.042 (1.034, 1.050)	1.036 (1.030, 1.043)	1.048 (1.041, 1.054)

*RR*: Risk Ratio, *CI*: Confidence Interval

7,

**Table 8:** The association between cumulative disinfection by products ^a^ (µg/l) exposure and colorectal cancer diagnosis in California during the years 2010 to 2015

Characteristics	Lag 0–1	Lag 0–5	Lag 0–10	Lag 0–15	Lag 0–20
	RRs (95% CI)	RRs (95% CI)	RRs (95% CI)	RRs (95% CI)	RRs (95% CI)
Total	0.987 (0.967, 1.007)	0.974 (0.973, 0.975)	0.966 (0.965, 0.966)	0.961 (0.960, 0.962)	0.961 (0.960, 0.962)
*Sex*					
Female	0.987 (0.957, 1.017)	0.974 (0.973, 0.975)	0.966 (0.965, 0.967)	0.961 (0.960, 0.963)	0.962 (0.960, 0.963)
Male	0.989 (0.979, 0.998)	0.976 (0.975, 0.977)	0.968 (0.967, 0.969)	0.962 (0.961, 0.964)	0.962 (0.961, 0.964)
*Race*					
White	0.991 (0.989, 0.993)	0.986 (0.985, 0.987)	0.984 (0.983, 0.985)	0.982 (0.981, 0.983)	0.983 (0.982, 0.984)
Black	0.995 (0.993, 0.997)	0.977 (0.973, 0.980)	0.954 (0.950, 0.959)	0.935 (0.929, 0.941)	0.935 (0.929, 0.941)
Hispanic	0.992 (0.990, 0.994)	0.965 (0.962, 0.969)	0.951 (0.949, 0.953)	0.944 (0.941, 0.946)	0.944 (0.942, 0.947)
Others	0.993 (0.991, 0.995)	0.979 (0.978, 0.981)	0.963 (0.961, 0.965)	0.952 (0.950, 0.955)	0.949 (0.946, 0.952)
*Age*					
< = 40	0.998 (0.997, 0.999)	0.992 (0.990, 0.994)	0.987 (0.984, 0.989)	0.984 (0.981, 0.987)	0.983 (0.979, 0.987)
41–64	0.988 (0.976, 1.001)	0.976 (0.975, 0.977)	0.966 (0.965, 0.968)	0.961 (0.959, 0.962)	0.961 (0.959, 0.962)
65–90	0.987 (0.962, 1.013)	0.974 (0.972, 0.975)	0.966 (0.965, 0.967)	0.961 (0.960, 0.963)	0.962 (0.960, 0.963)
> 90	0.992 (0.986, 0.998)	0.988 (0.984, 0.991)	0.980 (0.977, 0.984)	0.977 (0.973, 0.981)	0.976 (0.971, 0.981)

^a^Disinfection by products: sum of THMs (chloroform, bromodichloromethane, chlorodibromomethane and bromoform) and HAA5 (monochloroacetic acid, dichloroacetic acid (DCA), trichloroacetic acid (TCA), monobromoacetic acid, and dibromoacetic acid)

*RR*: Risk Ratio, *CI*: Confidence Interval

8,

**Table 9:** The incidence rate of colorectal cancer (i. e. All Ages, All Races/Ethnicities, Male and Female) from 2011–2015 Rate per 100,000 people

County	Age adjusted rate	Case count	Population
San Bernardino County	41.9	3846	10,456,617
Lake County	41.8	194	320,505
Butte County	40.5	566	1,112,508
Stanislaus County	40	1026	2,629,316
Sacramento County	39.6	2988	7,317,042
San Joaquin County	38.4	1310	3,535,533
Contra Costa County	38.3	2358	5,470,625
Riverside County	37.7	4427	11,467,864
Los Angeles County	37	18,657	50,036,337
Solano County	36.7	844	2,124,261
Kern County	36	1312	4,321,381
San Diego County	35	5766	16,089,725
Alameda County	34.9	2859	7,910,402
Fresno County	34.9	1520	4,776,383
Santa Clara County	34.5	3285	9,318,746
Santa Barbara County	34.1	801	2,176,804
Kings County	33.9	208	753,417
Ventura County	33.8	1534	4,196,521
San Luis Obispo	33.3	592	1,381,691
Calaveras County	33.2	122	223,872
Placer County	33.2	784	1,829,105
Marin County	32.8	605	1,291,597
Nevada County	32.6	239	492,858
San Mateo County	32.6	1429	3,737,863
Santa Cruz County	31.2	460	1,344,877
Total	897.9	57,732	154,315,850

Note: sorted from largest to smallest age adjusted rate

Source: https://gis.cdc.gov/Cancer/USCS/DataViz.html

9,

**Table 10:** The association between cumulative nitrate (mg/l as NO_3_) exposure and colorectal cancer diagnosis in California by stratifying for counties

Characteristics	Lag 0–1	Lag 0–5	Lag 0–10	Lag 0–15	Lag 0–20
	RRs (95% CI)	RRs (95% CI)	RRs (95% CI)	RRs (95% CI)	RRs (95% CI)
County					
Alameda	0.975 (0.972, 0.978)	0.897 (0.887, 0.907)	1.024 (0.990, 1.060)	0.940 (0.911, 0.970)	0.824 (0.804, 0.845)
Butte	1.005 (0.995, 1.016)	1.027 (0.977, 1.079)	1.136 (0.964, 1.339)	1.155 (0.884, 1.511)	1.165 (0.963, 1.408)
Calaveras	0.912 (0.416, 2.002)	1.742 (0.988, 3.073)	0.892 (0.793, 1.004)	2.61 (0.184, 5.001)	0.208 (0.009, 4.724)
Contra Costa	1.060 (1.052, 1.068)	1.095 (1.079, 1.110)	NA	NA	NA
Fresno	0.959 (0.948, 0.970)	1.147 (1.136, 1.158)	1.290 (1.248, 1.334)	1.100 (1.071, 1.130)	0.680 (0.654, 0.706)
Kern	1.110 (1.099, 1.120)	1.030 (1.015, 1.045)	0.988 (0.959, 1.017)	0.999 (0.980, 1.018)	0.989 (0.968, 1.009)
Kings	1.002 (0.978, 1.027)	0.978 (0.928, 1.032)	0.927 (0.877, 0.980)	0.881 (0.805, 0.965)	0.563 (0.414, 0.766)
Lake County	1.108 (1.045, 1.176)	1.099 (1.006, 1.200)	1.209 (1.019, 1.434)	1.147 (1.035, 1.271)	0.984 (0.851, 1.137)
Los Angeles	0.918 (0.916, 0.920)	1.286 (1.278, 1.295)	1.390 (1.373, 1.407)	1.190 (1.186, 1.194)	1.511 (1.485, 1.539)
Marin	1.019 (1.012, 1.027)	0.949 (0.925, 0.975)	1.006 (0.954, 1.061)	0.935 (0.850, 1.028)	0.933 (0.825, 1.055)
Nevada	1.058 (0.831, 1.346)	1.118 (0.855, 1.461)	0.792 (0.585, 1.070)	0.985 (0.915, 1.062)	0.942 (0.883, 1.005)
Placer	0.996 (0.988, 1.004)	0.971 (0.946, 0.998)	1.061 (0.990, 1.138)	1.147 (1.026, 1.283)	0.806 (0.629, 1.033)
Riverside	1.034 (1.028, 1.039)	1.135 (1.127, 1.143)	0.964 (0.953, 0.975)	1.084 (1.049, 1.121)	0.953 (0.940, 0.966)
Sacramento	1.012 (1.007, 1.016)	0.999 (0.999, 1.002)	0.908 (0.808, 1.008)	0.988 (0.887, 1.088)	0.903 (0.800, 1.003)
San Bernardino	1.027 (1.019, 1.035)	1.077 (1.066, 1.088)	1.010 (1.003, 1.017)	1.030 (1.019, 1.040)	0.974 (0.963, 0.985)
San Diego	1.010 (1.007, 1.012)	0.963 (0.962, 0.964)	0.951 (0.950, 0.952)	0.946 (0.943, 0.948)	0.907 (0.895, 0.920)
San Joaquin	0.984 (0.982, 0.987)	1.011 (1.000, 1.022)	0.978 (0.956, 1.001)	1.041 (0.964, 1.124)	0.776 (0.674, 0.892)
San Luis Obispo	0.984 (0.976, 0.993)	1.158 (0.800, 1.675)	NA	NA	NA
San Mateo	1.036 (1.017, 1.056)	1.155 (1.109, 1.203)	1.196 (1.173, 1.219)	1.192 (1.170, 1.215)	0.566 (0.523, 0.611)
Santa Barbara	1.020 (1.012, 1.029)	1.124 (1.105, 1.144)	1.074 (1.044, 1.104)	1.056 (1.028, 1.085)	1.072 (1.044, 1.101)
Santa Clara	0.994 (0.993, 0.996)	1.158 (1.146, 1.170)	0.947 (0.933, 0.962)	0.842 (0.832, 0.852)	0.794 (0.783, 0.806)
Santa Cruz	0.971 (0.943, 0.999)	0.840 (0.676, 1.045)	0.386 (0.257, 0.580)	0.923 (0.896, 0.951)	0.670 (0.282, 1.595)
Solano	1.014 (1.006, 1.022)	0.938 (0.904, 0.974)	1.058 (1.008, 1.111)	0.658 (0.571, 0.758)	0.596 (0.532, 0.669)
Stanislaus	1.013 (0.999, 1.028)	1.057 (1.034, 1.081)	1.016 (0.998, 1.033)	0.842 (0.810, 0.875)	0.802 (0.714, 0.901)
Ventura	1.000 (0.997, 1.003)	1.063 (1.051, 1.074)	1.114 (1.095, 1.134)	1.163 (1.101, 1.230)	0.550 (0.498, 0.608)

Lag 0–1: The one year mean concentration of Nitrate (mg/l as NO3) prior to colorectal cancer diagnosis confirmation; Lag 0–5: The five years mean concentration of Nitrate (mg/l as NO3) prior to colorectal cancer diagnosis confirmation; Lag 0–10: The ten years mean concentration of Nitrate (mg/l as NO3) prior to colorectal cancer diagnosis confirmation; Lag 0–15: The fifteen years mean concentration of Nitrate (mg/l as NO3) prior to colorectal cancer diagnosis confirmation; Lag 0–20: The twenty years mean concentration of Nitrate (mg/l as NO3) prior to colorectal cancer diagnosis confirmation; RR: Risk Ratio; CI: Confidence Interval

10

See Figs. 3, 4
